# Three New Species of *Clonostachys* (Hypocreales, Ascomycota) from China

**DOI:** 10.3390/jof8101027

**Published:** 2022-09-28

**Authors:** Zhao-Qing Zeng, Wen-Ying Zhuang

**Affiliations:** State Key Laboratory of Mycology, Institute of Microbiology, Chinese Academy of Sciences, Beijing 100101, China

**Keywords:** Bionectriaceae, morphology, sequence analyses, taxonomy

## Abstract

Three new species of *Clonostachys* are introduced based on specimens collected from China. *Clonostachys chongqingensis* sp. nov. is distinguished by pale yellow to pale orange-yellow perithecia with a very low papilla, clavate to subcylindrical asci possessing ellipsoidal to elongate-ellipsoidal spinulose ascospores 13–16 × 4.5–5.5 μm; it has acremonium- to verticillium-like conidiophores and ellipsoidal to rod-shaped conidia. *Clonostachys leptoderma* sp. nov. has pinkish-white subglobose to globose perithecia on a well-developed stroma and with a thin perithecial wall, clavate to subcylindrical asci with ellipsoidal to elongate-ellipsoidal spinulose ascospores 7.5–11 × 2.5–3.5 μm; it produces verticillium-like conidiophores and ellipsoidal to subellipsoidal conidia. *Clonostachys oligospora* sp. nov. features solitary to gregarious perithecia with a papilla, clavate asci containing 6–8 smooth-walled ascospores 9–17 × 3–5.5 μm; it forms verticillium-like conidiophores and sparse, subfusiform conidia. The morphological characteristics and phylogenetic analyses of combined nuclear ribosomal DNA ITS1-5.8S-ITS2 and beta-tubulin sequences support their placement in *Clonostachys* and their classification as new to science. Distinctions between the novel taxa and their close relatives are compared herein.

## 1. Introduction

*Clonostachys* Corda, typified by *C. araucaria* Corda, is characterized by solitary to gregarious, subglobose or globose to ovoid perithecia that are white, yellow, pale orange, tan, or brown; perithecial walls are KOH− and LA−; there are narrowly clavate to clavate asci containing eight ascospores; it produces penicillium-, verticillium-, gliocladium-, or acremonium-like conidiophores, cylindrical to narrowly flask-shaped phialides, and ellipsoidal to subfusiform conidia [1]. Members of the genus usually have a broad range of lifestyles and occur on the bark of recently dead trees, decaying leaves, and less frequently on other fungi, nematodes, and insects [1,2,3]. They are economically important in the fields of pharmaceutics and agriculture [4]. For instance, the secondary metabolites produced by *C. byssicola* Schroers exhibited antibacterial activities [5], and strains of *C. rosea* (Link) Schroers, Samuels, Seifert & W. Gams have been widely used as biocontrol agents [6].

About 100 names have been created under the genus *Clonostachys* (www.indexfungorum.org (accessed on 1 July 2022)), among which 65 species are commonly accepted [2,3,7,8,9,10,11,12,13,14,15,16,17,18,19,20]. Twenty-four species are known from China [2,7,21]. In this study, three additional taxa are introduced based on morphological characteristics and sequence analyses of combined nuclear ribosomal DNA ITS1-5.8S-ITS2 (ITS) and beta-tubulin (BenA) regions. Comparisons between these novel species and their close relatives are performed.

## 2. Materials and Methods

### 2.1. Sampling and Morphological Studies

Specimens were collected from Chongqing and Yunnan Province and were deposited in the Herbarium Mycologicum Academiae Sinicae (HMAS). Cultures were obtained by single ascospore isolation from fresh perithecium and are preserved in the State Key Laboratory of Mycology, Institute of Microbiology, Chinese Academy of Sciences. The methods of Hirooka et al. [22] were generally followed for morphological observations. Perithecial wall reactions were tested in 3% potassium hydroxide (KOH) and 100% lactic acid (LA). Longitudinal sections through the perithecia were made with a freezing microtome (YD-1508-III, Jinhua, China) at a thickness of 6–8 μm. Photographs were taken using a Canon G5 digital camera (Tokyo, Japan) connected to a Zeiss Axioskop 2 plus microscope (Göttingen, Germany). For colony characteristics and growth rates, strains were grown on potato dextrose agar (PDA) (200 g potato + 2% (*w*/*v*) dextrose + 2% (*w*/*v*) agar) and synthetic low-nutrient agar (SNA) [23] in 90 mm plastic Petri dishes at 25 °C for 2 weeks with alternating periods of light and darkness (12 h/12 h).

### 2.2. DNA Extraction, PCR Amplification, and Sequencing

The genomic DNA was extracted from fresh mycelium following the method of Wang and Zhuang [24]. Five primer pairs, namely ITS5/ITS4 [25], T1/T22 [26], acl1-230up/acl1-1220low [27], Crpb1a/rpb1c [28], and EF1-728F/EF2 [29,30], were used to amplify the sequences of ITS, BenA, ATP citrate lyase (ACL1), the largest subunit of RNA polymerase II (RPB1), and translation elongation factor 1-α (TEF1), respectively. PCR reactions were performed on an ABI 2720 Thermal Cycler (Applied Biosciences, Foster City, CA, USA) with a 25 μL reaction system consisting of 12.5 μL Taq MasterMix, 1 μL of each primer (10 μM), 1 μL template DNA, and 9.5 μL ddH_2_O. DNA sequencing was carried out in both directions with the same primer pairs using an ABI 3730 XL DNA Sequencer (Applied Biosciences, Foster City, CA, USA). Newly achieved sequences and those retrieved from GenBank are listed in Table 1. *Fusarium acutatum* Nirenberg & O’Donnell and *Nectria cinnabarina* (Tode) Fr. were chosen as outgroup taxa.

### 2.3. Sequence Alignment and Phylogenetic Analyses

Sequences were assembled and aligned with BioEdit 7.0.5 [31] and converted to nexus files by ClustalX 1.8 [32]. To confirm the taxonomic positions of the new species, ITS and BenA sequences were combined and analyzed with Bayesian inference (BI), maximum likelihood (ML), and maximum parsimony (MP) methods. A partition homogeneity test (PHT) was performed with 1000 replicates in PAUP*4.0b10 [33] to evaluate the statistical congruence between the two loci. The BI analysis was conducted by MrBayes 3.1.2 [34] using a Markov chain Monte Carlo (MCMC) algorithm. Nucleotide substitution models were determined by MrModeltest 2.3 [35]. Four Markov chains were run simultaneously for 1,000,000 generations with the trees sampled every 100 generations. A 50% majority rule consensus tree was computed after excluding the first 2500 trees as “burn-in”. Bayesian inference posterior probability (BIPP) was determined from the remaining trees. The ML analysis was performed via IQ-Tree 1.6.12 [36] using the best model for each locus, as chosen by ModelFinder [37]. The MP analysis was performed with PAUP 4.0b10 [33] using heuristic searches with 1000 replicates of random addition of sequences and subsequent TBR (tree bisection and reconnection) branch swapping. The topological confidence of the resulting trees and statistical support of the branches were tested in maximum parsimony bootstrap proportion (MPBP) with 1000 replications and each with 10 replicates of the random addition of taxa. Trees were examined by TreeView 1.6.6 [38]. The maximum likelihood bootstrap (MLBP) values, MPBP values greater than 70%, and BIPP values greater than 90% were shown at the nodes.

## 3. Results

### 3.1. Phylogeny

**Figure 1:**
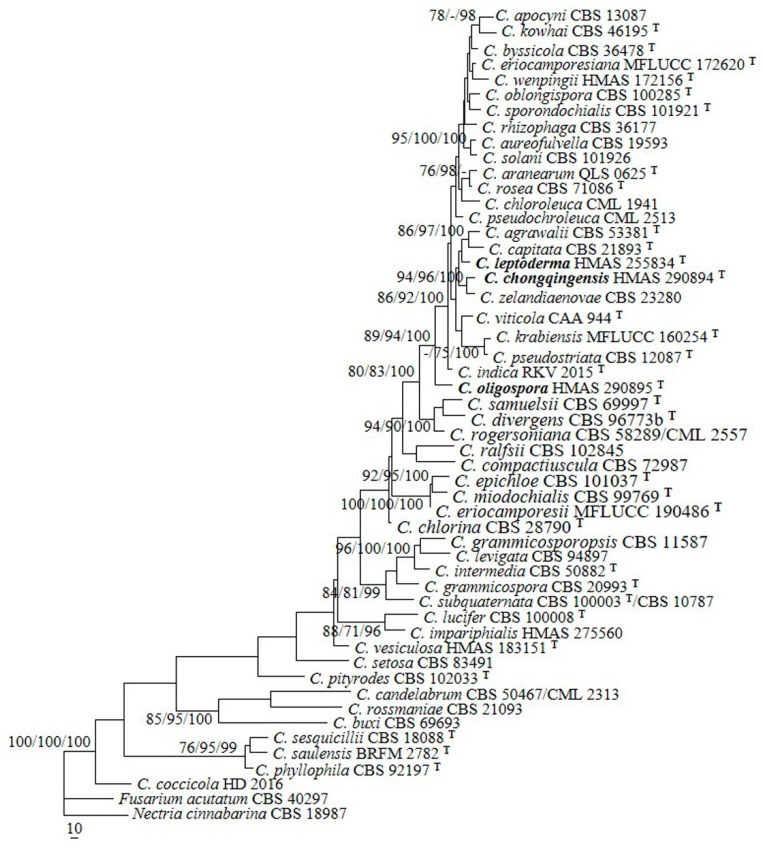
A maximum parsimony tree generated from analyses of combined ITS and BenA sequences of *Clonostachys* species. The MP analysis was performed using heuristic searches with 1000 replicates. MPBP (**left**) and MLBP (**middle**) values greater than 70% and BIPP (**right**) values greater than 90% were shown at the nodes. *Fusarium acutatum* and *Nectria cinnabarina* were chosen as outgroup taxa.

The sequences of ITS and BenA from 50 representative species of *Clonostachys* were analyzed. The PHT (*p* = 0.05) indicated that the individual partitions were not highly incongruent [39]; thus, the two loci were combined for phylogenetic analyses. In the MP analysis, the datasets included 1159 nucleotide characters, of which 543 bp were constant, 154 were variable and parsimony-uninformative, and 462 were parsimony-informative. The MP analysis resulted in 123 most parsimonious trees (tree length = 2794, consistency index = 0.4098, homoplasy index = 0.5902, retention index = 0.4677, rescaled consistency index = 0.1917). One of the MP trees generated is shown in Figure 1. The topologies of the BI and ML trees were similar to that of the MP tree. The isolates 12581, 12672, and 11691 were grouped with the other *Clonostachys* taxa investigated (MPBP/MLBP/BIPP = 100%/100%/100%), which confirmed their taxonomic positions. The isolate 12581 was grouped with *C. agrawalii* (Kushwaha) Schroers and *C. capitata* Schroers, with low statistical support. The isolate 12672 clustered with *C. zelandiaenovae* Schroers (MPBP/MLBP/BIPP = 94%/96%/100%), and 11691 formed a separate lineage.

### 3.2. Taxonomy

**Figure 2:**
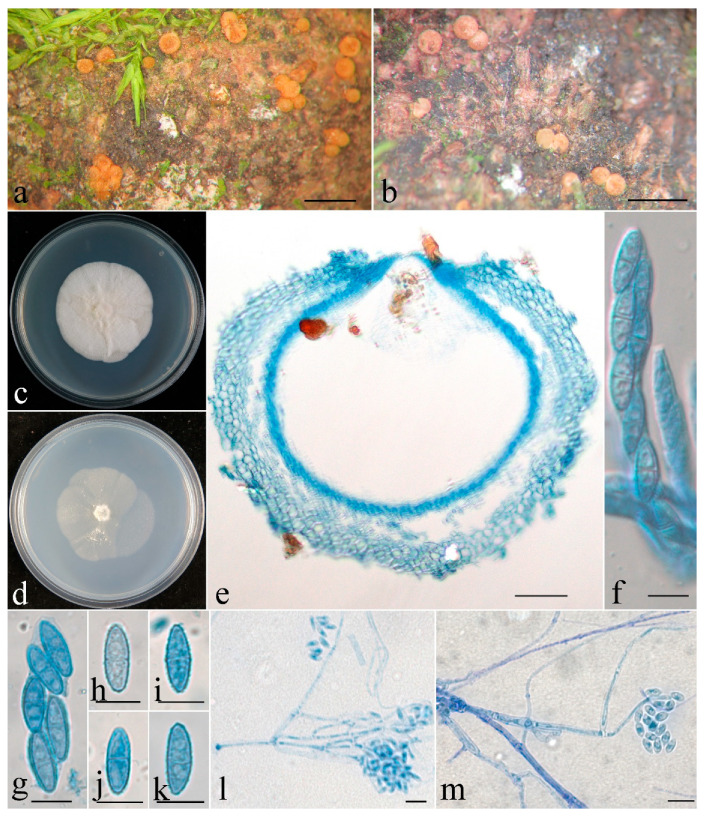
*Clonostachys chongqingensis* (holotype). (**a**,**b**) Ascomata on natural substratum; (**c**) colony after 2 weeks at 25 °C on PDA; (**d**) colony after 2 weeks at 25 °C on SNA; (**e**) median section through the perithecium; (**f**) asci with ascospores; (**g**–**k**) ascospore; (**l**,**m**) conidiophores, phialides, and conidia. Bars: (**a**,**b**) = 1 mm; (**e**) = 50 μm; (**f**–**m**) = 10 μm.

***Clonostachys chongqingensis*** Z.Q. Zeng and W.Y. Zhuang, sp. nov. (Figure 2).

Fungal Names: FN571276.

**Etymology**: The specific epithet refers to the type locality of the fungus.

**Typification**: China, Chongqing City, Jinfo Mountain, 29°2′50″ N 107°11′0″ E, on rotten bark of *Alnus* sp., 25 October 2020, Z.Q. Zeng, H.D. Zheng, X.C. Wang, C. Liu 12672 (holotype HMAS 290894).

**DNA barcodes**: ITS OP205475, BenA OP205324, ACL1 OP493559, TEF1 OP493562.

The mycelium was not visible on the natural substratum. Perithecia were superficial, solitary to gregarious, non-stromatic or with a basal stroma, subglobose to globose, with very low papilla and slightly roughened surface; they mostly did not collapse upon drying, and a few were slightly pinched at the apical portion, colored pale yellow to pale orange-yellow. There was no color change in 3% KOH or 100% LA, and the size was 304–353 × 294–392 μm. Perithecial walls were two-layered, 40–70 μm thick; the outer layer was of textura globulosa to textura angularis, 30–45 μm thick, with cells 5–15 × 3–12 μm and cell walls 0.8–1 μm thick. The inner layer was of textura prismatica, 10–25 μm thick, with cells 8–14 × 2.5–3.5 μm and cell walls 1–1.2 μm thick. Asci were clavate to subcylindrical, eight-spored, with a round and simple apex, and 60–85 × 6–13 μm. Ascospores were ellipsoidal to elongate-ellipsoidal, uniseptate, hyaline, spinulose, and uniseriate or irregular-biseriate, and 13–16 × 4.5–5.5 μm.

Colonies on PDA were 53 mm in diam. in average after 2 weeks at 25 °C; their surface was cottony, with a dense, whitish aerial mycelium. Colonies on SNA were 50 mm in diam. in average after 2 weeks at 25 °C; their surface was velvet, with a sparse, whitish aerial mycelium. Conidiophores were acremonium- to verticillium-like, arising from aerial hyphae and septate. Phialides were subulate tapered toward the apex, 15–74 μm long, 1.6–2.5 μm wide at the base, and 0.3–0.4 μm wide at the tip. Conidia were ellipsoidal to rod-shaped, unicellular, smooth-walled, hyaline, and 4–10 × 2.5–4 μm.

**Notes**: Morphologically, the species most resembles *C. sesquicillii* Schroers in having superficial, solitary to gregarious ascomata and clavate to subcylindrical asci with eight ellipsoidal, single-septate, spinulose ascospores [1]. However, the perithecia of the latter are often laterally or apically pinched when dry and have shorter asci (35–63 μm long) and smaller ascospores (8.2–14.4 × 2.2–4.4 μm) [1]. The two-locus phylogeny indicated the two fungi are remotely related (Figure 1).

Phylogenetically, *C. chongqingensis* is closely related to *C. zelandiaenovae* (Figure 1). The latter differs in its well-developed stroma, narrowly clavate asci with an apex ring, and wider ascospores (3.8–7.4 μm wide) [1].

**Figure 3:**
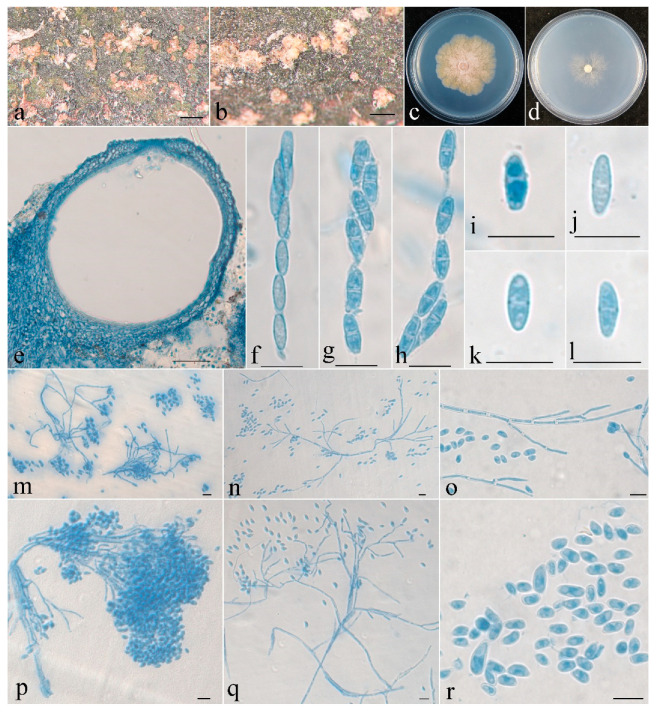
*Clonostachys leptoderma* (holotype). (**a**,**b**) Ascomata on natural substratum; (**c**) colony after 2 weeks at 25 °C on PDA; (**d**) colony after 2 weeks at 25 °C on SNA; (**e**) median section through the perithecium; (**f**–**h**) asci with ascospores; (**i**–**l**) ascospore; (**m**–**q**) conidiophores, phialides, and conidia; (**r**) conidia. Bars: (**a**,**b**) = 1 mm; (**e**) = 50 μm; (**f**–**r**) = 10 μm.

***Clonostachys leptoderma*** Z.Q. Zeng and W.Y. Zhuang, sp. nov. (Figure 3)

Fungal Names: FN571277.

**Etymology**: The specific epithet refers to the thin-walled perithecia.

**Typification**: China, Chongqing City, Jinyun Mountain, 29°49′46″ N 106°22′49″ E, on rotten bark, 23 October 2020, Z.Q. Zeng, H.D. Zheng, X.C. Wang, C. Liu 12581 (holotype HMAS 255834).

**DNA barcodes**: ITS OP205474, BenA OP205323, RPB1 OP493564, TEF1 OP493561.

The mycelium was not visible on the natural substratum. Perithecia were superficial, solitary to gregarious, with a well-developed stroma, subglobose to globose, non-papillate, with surface slightly roughened, and did not collapse upon drying. They were pinkish-white, did not change color in 3% KOH or 100% LA, and were with a size of 216–284 × 206–265 μm. Perithecial walls were two-layered, 13–45 μm thick; the outer layer was of textura globulosa to textura angularis, 8–23 μm thick, with cells 5–10 × 4–8 μm and cell walls 1–1.2 μm thick; the inner layer was of textura prismatica, 5–22 μm thick, with cells 5–12 × 2–3 μm and cell walls 0.8–1 μm thick. Asci were clavate to subcylindrical, 6–8-spored, with a round and simple apex, and 53–63 × 4.8–7 μm. Ascospores were ellipsoidal to elongate-ellipsoidal, uniseptate, hyaline, spinulose, uniseriate or irregular-biseriate, and 7.5–11 × 2.5–3.5 μm.

Colonies on PDA was 31 mm in diam. in average after 2 weeks at 25 °C; their surface was cottony, with a dense, whitish aerial mycelium, and it produced a yellowish-brown pigment in medium. Colony on SNA were 18 mm in diam. in average after 2 weeks at 25 °C, with a sparse, whitish aerial mycelium. Conidiophores were verticillium-like, arising from aerial hyphae; they were septate, with dense phialides. Phialides were subulate, tapering toward the apex, 9–18 μm long, 1.5–2.5 μm wide at the base, and 0.2–0.3 μm wide at the tip. Conidia were subglobose, ellipsoidal to subellipsoidal, unicellular, smooth-walled, hyaline, and 2–7 × 2–5 μm.

**Notes**: Morphologically, the fungus is most similar to *C. epichloe* Schroers in having solitary to gregarious perithecia and ellipsoidal, bi-cellular, spinulose ascospores of a similar size [1]. Nevertheless, the latter differs in its smaller perithecia (140–240 × 140–200 μm) that is pinched when dry, its wider asci (5–10 μm wide) [1], and the presence of 36 bp and 132 bp divergences in the ITS and BenA regions. Obviously, they are not conspecific.

Phylogenetically, *C. leptoderma* is closely related to *C. capitata* and *C. agrawalii* (Figure 1). *Clonostachys capitata* can be differentiated by its thicker perithecial wall (45–60 μm thick), wider asci (7–12 μm wide), and larger ascospores (11.6–18.8 × 3.6–5.8 μm) [1]. *Clonostachys agrawalii*, which is known to have only an asexual stage, can be easily distinguished by its bi- to quarter-verticillate conidiophores and its cylindrical to flask-shaped, somewhat larger phialides (7–42 × 1.4–3.4 μm) [1].

**Figure 4:**
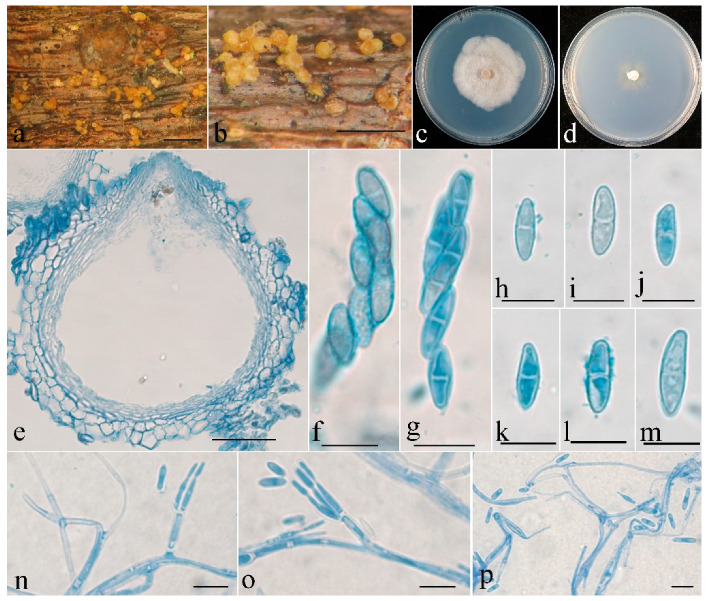
*Clonostachysoligospora* (holotype). (**a**,**b**) Ascomata on natural substratum; (**c**) colony after 2 weeks at 25 °C on PDA; (**d**) colony after 2 weeks at 25 °C on SNA; (**e**) median section through the perithecium; (**f**,**g**) asci with ascospores; (**h**–**m**) ascospore; (**n**–**p**) conidiophores, phialides, and conidia. Bars: (**a**,**b**) = 1 mm; (**e**) = 50 μm; (**f**–**p**) = 10 μm.

***Clonostachys oligospora*** Z.Q. Zeng and W.Y. Zhuang, sp. nov. (Figure 4)

Fungal Names: FN571278.

**Etymology**: The specific epithet refers to the very few conidia produced.

**Typification**: China, Yunnan Province, Chuxiong Prefecture, Zixi Mountain, Xianrengu, 25°54′0″ N 101°24′46″ E, on a rotten twig, 23 September 2017, Y. Zhang, H.D. Zheng, X.C. Wang, Y.B. Zhang 11691 (holotype HMAS 290895).

**DNA barcodes**: ITS OP205473, BenA OP205322, ACL1 OP493560, RPB1 OP493563.

The mycelium was not visible on the natural substratum. Perithecia were superficial, solitary to gregarious, either with a basal stroma or non-stromatic. They were subglobose to globose and papillate, with surface slightly warted; the warts were 6–25 μm high. They did not collapse upon drying, were colored pale yellow to light yellow, did not change color in 3% KOH or 100% LA, and were 225–274 × 225–265 μm. The perithecial walls were two-layered, 25–48 μm thick; the outer layer was of textura globulosa to textura angularis, 15–38 μm thick, with cells 8–18 × 9–15 μm and cell walls 0.5–0.8 μm thick; the inner layer was of textura prismatica, 10–15 μm thick, with cells 5–8 × 1.5–2.5 μm and cell walls 0.8–1 μm thick. Asci were clavate, 6–8-spored, with a rounded and simple apex, and 45–65 × 7.5–11 μm. Ascospores were ellipsoidal, uniseptate, hyaline, smooth-walled, uniseriate or irregular biseriate, and 9–17 × 3–5.5 μm.

Colonies on PDA were 50 mm in diam. in average after 2 weeks at 25 °C; their surface was cottony, with a dense, whitish aerial mycelium. Colonies on SNA were 25 mm in diam. in average after 2 weeks at 25 °C, with a sparse, whitish aerial mycelium. Conidiophores were verticillium-like, arising from aerial hyphae, and septate. Phialides were subulate, tapering toward the apex, 9–15 μm long; they were 1.5–2.5 μm wide at the base and 0.2–0.3 μm wide at the tip. Conidia were sparse, subfusiform, unicellular, smooth-walled, hyaline, and 5–13 × 1.8–2.2 μm.

**Notes**: Among the known species of *Clonostachys*, this fungus resembles *C. setosa* (Vittal) Schroers in terms of solitary to gregarious perithecia and ascospores that are ellipsoidal, bi-cellular, smooth-walled, and of a similar size [1]. However, the latter fungus is distinguished by asci with an apical ring, as well as by conidiophores that are penicillium-like and cylindrical conidia that are slightly larger (8.6–19.2 × 2–3.2 μm) [1]. In addition, there are 47 bp and 128 bp divergences in the ITS and BenA regions between HMAS 290895 and CBS 834.91. Both morphology and DNA sequence data support their distinction at the species level.

## 4. Discussion

Although *Clonostachys* was established in 1839, the name was not commonly used until Schroers’ monographic treatment of the genus, from which 44 species were accepted [1]. The generic name *Bionectria* Speg. was introduced later [40], and the genus was reviewed by Rossman et al. [41]; in that work, the species included those previously placed in the *Nectria ochroleuca* group, the *N. ralfsii* group, and the *N. muscivora* group, as well as those having *Sesquicillium* W. Gams asexual stages. *Clonostachys* and *Bionectria* are of anamorph and teleomorph connections [1,41]. According to the current International Code of Nomenclature for algae, fungi, and plants [42], under the principle that one fungus requires one name, *Clonostachys* was recommended as the preferable name [43].

The previous phylogenetic overview of *Clonostachys* that was based on two-locus (ITS and BenA) sequence analyses showed that the genus is monophyletic [19,20]. Our analyses provided a similar tree topology, and species of the genus formed a well-supported clade (MPBP/MLBP/BIPP = 100%/100%/100%), including the three new taxa (Figure 1). *Clonostachys oligospora* is a well-separated lineage in between *C. indica* Prasher & R. Chauhan and *C. samuelsii* Schroers. *Clonostachys chongqingensis* clustered with *C. zelandiaenovae*, receiving relatively high statistical support (MPBP/MLBP/BIPP = 94%/96%/100%), and had moderate sequence divergences, i.e., 11/518 bp (2.1%) for ITS and 15/557 bp (2.7%) for BenA. *Clonostachys leptoderma* was grouped with *C. agrawalii* and *C. capitata*, which is poorly supported. Compared with the previously demonstrated phylogenies [19,20], minor changes were detected. For example, *C. pseudostriata* Schroers formerly constituted a separate lineage by itself [19,20]; whereas, with the joining of the new species, the fungus seemed to be closely related to *C. krabiensis* Tibpromma & K.D. Hyde and *C. viticola* C. Torcato & A. Alves, with low statistical support. Comparisons between each new species and closely related taxa are provided in Table 2. Along with the discovery of additional new species, the relationships among the species of the genus will become well-established.

More than 220 secondary metabolites have been reported from species of the genus. For example, *C. byssicola*, *C. candelabrum* (Bonord.) Schroers, *C. compactiuscula* (Sacc.) D. Hawksw. & W. Gams, *C. grammicospora* Schroers & Samuels, *C. pityrodes* Schroers, *C. rogersoniana* Schroers, and *C. rosea* were demonstrated to have the potential for biocontrol application [4,44,45,46,47]. Meanwhile, strains of *C. rosea* were occasionally reported as an opportunistic phytopathogen [48,49]. Therefore, studies on the biodiversity of *Clonostachys* are of theoretical and practical importance and need to be carried out continuously and extensively. China is diverse in climate, vegetation, and geographic structures and has rich niches for organisms [50,51]. Large-scale surveys in unexplored regions will significantly improve our knowledge of fungal species diversity.

## 5. Conclusions

The species diversity of the genus *Clonostachys* was investigated, and three new species were discovered. With the joining of the new species, the phylogenetic relationships among species of the genus are updated.

## Figures and Tables

**Table 1:** Sequences used in this study.

Species	Herbarium/Strain Numbers	GenBank Accession Numbers	
		ITS	BenA
*C. agrawalii*	CBS 53381	AF358241	AF358187
*C. apocyni*	CBS 13087	AF210688	AF358168
*C. aranearum*	QLS 0625	NR164542	KU212400
*C. aureofulvella*	CBS 19593	AF358226	AF358181
*C. buxi*	CBS 69693	KM231840	KM232111
*C. byssicola*	CBS 36478	MH861151	AF358153
*C. candelabrum*	CBS 50467/CML 2313	MH859044	KF871186
*C. capitata*	CBS 21893	AF358240	AF358188
*C. chlorina*	CBS 28790	NR137651	-
*C. chloroleuca*	CML 1941	KC806286	KF871172
*C.* *chongqingensis*	HMAS 290894	**OP205475 ^a^**	**OP205324**
*C. coccicola*	HD 2016	KU720552	KU720552
*C. compactiuscula*	CBS 72987	AF358242	AF358193
*C. divergens*	CBS 96773b	NR137532	AF358191
*C. epichloe*	CBS 101037	AF210675	AF358209
*C. eriocamporesiana*	MFLUCC 17-2620	NR168235	MN699965
*C. eriocamporesii*	MFLUCC 190486	NR168236	-
*C. grammicospora*	CBS 20993	NR137650	AF358206
*C. grammicosporopsis*	CBS 11587	AF210679	AF358204
*C. impariphialis*	HMAS 275560	KX096609	-
*C. indica*	RKV 2015	KT291441	KT291441
*C. intermedia*	CBS 50882	NR137652	AF358205
*C. kowhai*	CBS 46195	AF358250	AF358170
*C. krabiensis*	MFLUCC 160254	NR168189	-
*C. leptoderma*	HMAS 255834	**OP205474**	**OP205323**
*C. levigata*	CBS 94897	AF210680	AF358196
*C. lucifer*	CBS 100008	AF210683	AF358208
*C. miodochialis*	CBS 99769	NR137649	AF358210
*C. oblongispora*	CBS 100285	AF358248	AF358169
*C.* *oligospora*	HMAS 290895	**OP205473**	**OP205322**
*C. phyllophila*	CBS 92197	NR137531	-
*C. pityrodes*	CBS 102033	AF210672	AF358212
*C. pseudochroleuca*	CML 2513	KJ499909	KF871188
*C. pseudostriata*	CBS 12087	MH862056	AF358184
*C. ralfsii*	CBS 102845	AF358253	AF358219
*C. rhizophaga*	CBS 36177	AF358228	AF358158
*C. rogersoniana*	CBS 58289/CML 2557	AF210691	KX185047
*C. rosea*	CBS 71086	MH862010	AF358161
*C. rossmaniae*	CBS 21093	AF358227	AF358213
*C. samuelsii*	CBS 69997	AF358236	AF358190
*C. saulensis*	BRFM 2782	MK635054	-
*C. sesquicillii*	CBS 18088	AF210666	AF358214
*C. setosa*	CBS 83491	AF210670	AF358211
*C. solani*	CBS 101926	AF358230	AF358179
*C. sporondochialis*	CBS 101921	AF210685	AF358149
*C. subquaternata*	CBS 100003/CBS 10787	MT537603	AF358207
*C. vesiculosa*	HMAS 183151	NR119828	-
*C. viticola*	CAA 944	MK156282	MK156290
*C. wenpingii*	HMAS 172156	EF612465	HM054127
*C. zelandiaenovae*	CBS 23280	AF210684	AF358185
*F. acutatum*	CBS 40297	NR111142	KU603870
*N. cinnabarina*	CBS 18987	HM484699	HM484835

^a^ Numbers in bold indicate the newly provided sequences.

**Table 2:** Morphological comparisons of the new species and their close relatives.

Species	Sexual Morph			Asexual Morph			Reference Source
	Perithecia	Asci	Ascospores	Conidiophores	Phialides	Conidia	
*C. chongqingensis*	Subglobose to globose, 304–353 × 294–392 μm.	Clavate to subcylindrical, 60–85 × 6–13 μm.	Ellipsoidal to elongate-ellipsoidal, spinulose, 13–16 × 4.5–5.5 μm.	Acremonium- to verticillium-like.	Subulate, 15–74 × 1.6–2.5 μm.	Ellipsoidal to rod-shaped, 4–10 × 2.5–4 μm.	This study
*C. krabiensis*	N.A.	N.A.	N.A.	Aggregated into sporodochia.	Subulate, 10–13 × 1.5–2.5 μm.	Cylindrical to oblong, 5–7 × 1–2 μm.	[15]
*C. sesquicillii*	Globose, 250–300 μm diam.	Narrowly clavate to cylindrical, 35–63 × 5–13 μm.	Ellipsoidal, warted, 8.2–14.4 × 2.2–4.4 μm.	Penicillate conidiophores bi- to quarter-verticillate, verticillate conidiophores sparsely formed.	Cylindrical to flask-shaped, 6.4–18.8 × 1.4–3.6 μm.	Ellipsoidal to oblong ellipsoidal, 4.2–9.6 × 1.6–3 μm.	[1]
*C. viticola*	N.A.	N.A.	N.A.	Primary conidiophores verticillate, secondary conidiophores bi- to ter-verticillate, 45.3–64.7 × 2.1–3.7 μm.	Cylindrical, 10.4–32.8 × 1.7–2.7 μm.	Ellipsoidal to oval, 4.5–6.7 × 2.4–3.4 μm.	[3]
*C. zelandiaenovae*	Subglobose to globose, 290–550 μm diam.	Narrowly clavate, 60–104 × 7–15.5 μm.	Ellipsoidal, spinulose, rarely smooth, 11.6–21.4 × 3.8–7.4 μm.	Primary conidiophores verticillate, secondary conidiophores penicillate, ter- to quinquies-verticillate.	Cylindrical to narrowly flask-shaped, 4.8–20.6 × 1.6–3.4 μm.	Distally broadly rounded, 4–13.2 × 2.4–4.2 μm.	[1]
*C. leptoderma*	Subglobose to globose, 216–284 × 206–265 μm.	Clavate to subcylindrical, 53–63 × 4.8–7 μm.	Ellipsoidal to elongate-ellipsoidal, spinulose, 7.5–11 × 2.5–3.5 μm.	Verticillium-like.	Subulate, 9–18 × 1.5–2.5 μm.	Subglobose, ellipsoidal to subellipsoidal, 2–7 × 2–5 μm.	This study
*C. agrawalii*	N.A.	N.A.	N.A.	Primary conidiophores irregularly branched to ter-verticillate, secondary conidiophores bi- to quarter-verticillate.	Cylindrical to flask-shaped, 7–42 × 1.4–3.4 μm.	Ends broadly rounded, 3.8–5.8 × 2.2–3 μm.	[1]
*C. capitata*	Subglobose to oval, around 300 μm diam.	Narrowly clavate, 50.5–89.5 × 7–12 μm.	Ellipsoidal to oblong-ellipsoidal, spinulose to warted, 11.6–18.8 × 3.6–5.8 μm.	Primary conidiophores verticillium-like, secondary conidiophores ter- to quinquies-verticillium-like.	Cylindrical to narrowly flask-shaped, 8.8–46.6 × 1.4–3.6 μm.	Ends broadly rounded, 4.6–12.4 × 2.2–4.2 μm.	[1]
*C. epichloe*	140–240 × 140–200 μm.	Clavate to narrowly clavate, 32–65 × 5–10 μm.	Ellipsoidal, smooth to spinulose, 7.2–13 × 2.4–4.4 μm.	Divergently branched to adpressed.	Cylindrical to narrowly flask-shaped, 7–29 × 2.2–3.2 μm.	Ellipsoidal to narrowly clavate, 4.8–9.6 × 1.6–3.6 μm.	[1]
*C. oligospora*	Subglobose to globose, 225–274 × 225–265 μm.	Clavate, 45–65 × 7.5–11 μm.	Ellipsoidal, smooth, 9–17 × 3–5.5 μm.	Verticillium-like.	Subulate, 9–15 × 1.5–2.5 μm.	Sparse, subfusiform, 5–13 × 1.8–2.2 μm.	This study
* C. indica*	N.A.	N.A.	N.A.	Primary conidiophores verticillium-like, secondary conidiophores bi- to quarter-verticillate.	Cylindrical, 10.5–35.9 × 1.9–3.9 μm.	Ovoid to subglobose, 3.9–7.4 × 2–3.7 μm.	[12]
*C. samuelsii*	Subglobose to oval, 250–350 μm diam.	Narrowly clavate, 43–71 × 5–11.5 μm.	Ellipsoidal to broadly ellipsoidal, warted, 7.8–15.4 × 2.8–5.6 μm.	Penicillate or irregularly penicillate, ter- to quarter-verticillate.	Phialides cylindrical to narrowly flask-shaped, 3.8–20.6 × 1.8–5 μm.	Conidia ellipsoidal, 4.4–11.6 × 2.2–3.8 μm.	[1]
*C. setosa*	Globose, around 200 μm diam.	Narrowly clavate, 45–53 × 6.5–9 μm.	Ellipsoidal, smooth to striate, 8.8–13 × 2.4–3.8 μm.	Penicillate, mono- to ter-verticillate.	Cylindrical, 5.4–13.4 × 2.4–4.2 μm.	Cylindrical, 8.6–19.2 × 2–3.2 μm.	[1]

## Data Availability

The names of the new species were formally registered in the database Fungal Names (https://nmdc.cn/fungalnames (accessed on 11 August 2022)). Specimens were deposited in the Herbarium Mycologicum Academiae Sinicae (HMAS). The newly generated sequences were deposited in GenBank (https://www.ncbi.nlm.nih.gov/genbank (accessed on 22 September 2022)).
